# Effects of a 12-Week Exercise Training Program on Physical Function in Institutionalized Frail Elderly

**DOI:** 10.1155/2018/7218102

**Published:** 2018-01-11

**Authors:** Cristiane Batisti Ferreira, Pâmela dos Santos Teixeira, Geiane Alves dos Santos, Athila Teles Dantas Maya, Paula Americano do Brasil, Vinícius Carolino Souza, Cláudio Córdova, Aparecido Pimentel Ferreira, Ricardo Moreno Lima, Otávio de Toledo Nóbrega

**Affiliations:** ^1^Interdisciplinary Center for Research Integrated Colleges Promove Brasília, Brasília, Brazil; ^2^Medical Faculty, University of Brasília (UnB), Brasília, Brazil; ^3^Physical Education Program, Catholic University of Brasília (UCB-DF), Brasília, Brazil; ^4^Physical Education Program, University of Brasília (UnB), Brasília, Brazil

## Abstract

With the increase in life expectancy, the Brazilian elderly population has risen considerably. However, longevity is usually accompanied by problems such as the loss of functional capacity, cognitive decline, frailty syndrome, and deterioration in anthropometric parameters, particularly among those living in long-term care facilities. This randomized controlled trial aimed to verify the effects of exercise training on biochemical, inflammatory, and anthropometric indices and functional performance in institutionalized frail elderly. The sample consisted of 37 elderly people of both genders, aged 76.1 ± 7.7 years, who were randomly allocated into 2 groups: 13 individuals in the exercise group (EG) and 24 in the control group (CG). Anthropometrics, clinical history, functional tests, and biochemical evaluation were measured before and after the completion of a physical exercise program, which lasted for 12 weeks. The 12-week exercise program for frail elderly residents in a long-term care facility was efficient in improving muscle strength, speed, agility, and biochemical variables, with reversal of the frailty condition in a considerable number. However, no effects in anthropometric and inflammatory parameters were noted.

## 1. Introduction

Aging is a continuous process in which a progressive decline takes place in most physiological systems. This may expose the elderly to frailty, a syndrome characterized by a decrease in the homeostatic reserve and reduction of the body's ability to endure and perform, leading to a cumulative, vicious cycle of decline in multiple physiological systems and to vulnerability to adverse outcomes [1, 2].

In addition to physiological problems, aging is usually related to socioeconomic disadvantages. It is usually accompanied by an increase in expenditure, particularly with medications, in addition to decreasing income, since there is loss of production capacity and dependence almost exclusively of retirement income. On the other hand, the Brazilian family has changed significantly over the years, especially after the women's entry into the labor market, making home care difficult and increasing the demand for long-stay institutions. However, institutionalization may result in damage to functional decline and physical dependence, since older people living in the current models of long-term care facilities (LTCFs) do not seem to be properly stimulated [3].

Frailty decreases functional capacity by affecting the strength necessary to perform everyday activities [4] such as walking, raising from a chair, and reducing balance [5]. It is also insidiously related to a higher incidence of falls and disability as well as to hospitalization and mortality [1, 2, 6]. Consequently, frailty has healthcare cost implications, and its prevention could lead to a reduction in public costs and individual burden, which grows proportionally as the world population ages [7, 8]. Moreover, there is evidence that a decrease in physical capacity of older adults is related to an abnormal biochemical milieu such as insulin resistance, dyslipidemia, and systemic inflammation [9–12], but some controversy remains in this regard. Exercise training may represent a nonpharmacological strategy to prevent or treat frailty [13].

In terms of physical fitness, muscle strength seems to play a pivotal role in functional independence, with low muscle strength associated with a variety of comorbidities [14, 15] as well as with disability to perform everyday activities [16]. In addition to strength training, benefits for older adults have been observed in studies that evaluated multicomponent training programs [17–20]. However, an intervention capable of providing greater benefits has not been defined yet [20]. Also, physical exercise programs specific to the elderly population do not seem to be accessible, especially for institutionalized older people [18]. Thus, the aim of this study was to verify the effect of physical training on biochemical, inflammatory, and anthropometric traits as well as on functional performance of institutionalized frail elderly.

## 2. Materials and Methods

### 2.1. Study Characterization and Sample

**Figure 1:**
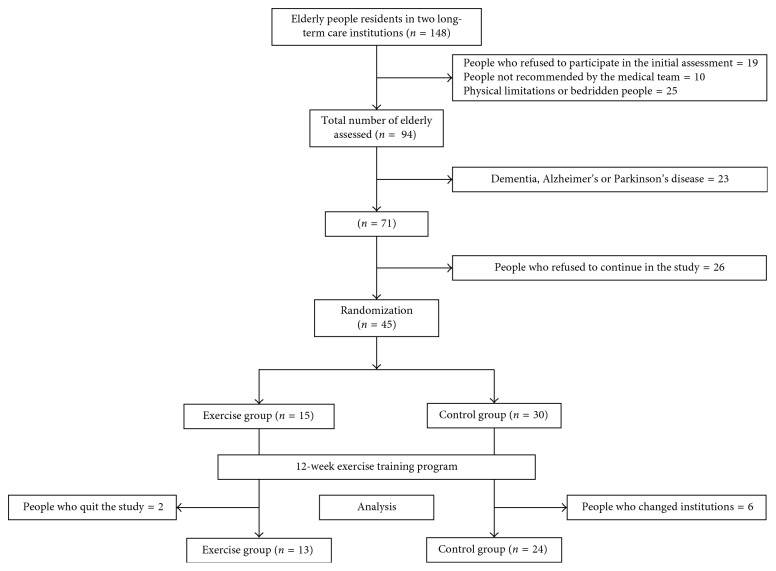
A schematic drawing of the sample.

This is a randomized experimental study conducted with institutionalized elderly volunteers. The sample consisted of 37 individuals of both genders, aged 60 years or older, who were residents of an LTCF in Brasília, Brazil. Subjects were randomly allocated to 2 groups after a series of recruitment steps as follows: 13 individuals in the exercise group (EG) and 24 in the control group (CG), according to a 2 : 1 ratio rationale between the CG and EG (Figure 1). Randomization was carried out by means of simple lottery applied to each subject in a process based on blindingly choosing cards which exhibited two options of letter (A or B), each representing one branch (CG or EG), with the amount of one letter in twofold excess compared to the other. The ratio of 2 : 1 between the CG and EG relies in the ethical premise of avoiding exposure of a vulnerable individual to a treatment arm of the study when efficacy is under consideration. This study was approved by the institutional research ethics committee, and the volunteers signed a consent form. Subjects aged over 60 years were diagnosed as prefrail or frail and who did not present limitations that precluded cognitive tests were recruited. Participants were declared fit by a medical doctor allowing them to undergo the exercise training program. The elderly diagnosed with dementia, Alzheimer's disease, or Parkinson's disease were excluded from the present study.

### 2.2. General Procedures of the Study

Experimental design of the study was based on data on anthropometric measurements, clinical history, functional tests, and biochemical evaluation assessed before and after the completion of a physical exercise program that lasted for 12 weeks.

The elderly in the EG participated in a training program performed 3 times a week, with sessions lasting 40 minutes, while subjects in the CG were instructed to maintain their usual activities of everyday life and to not change their physical activity habits during the study period.

### 2.3. Anthropometric Variables

To measure body mass, individuals stood on a Britannia® digital scale with a resolution of 0.1 kg wearing as few clothes as possible, immobile until the value was stable on the display. Height was assessed with a measuring tape with the subjects erected and arms relaxed, after deep inhalation. The body mass index (BMI) was determined as kg/m^2^. The conicity index was determined by measures of weight, height, and waist circumference using the following mathematical equation:

(1) $$\begin{array}{c} \text{conicity index}=\frac{\text{waist circumference}\left(\text{m}\right)}{\text{0.}109\sqrt{\text{body weight}\left(\text{kg}\right)/\text{height}\left(\text{m}\right)}}. \end{array}$$

### 2.4. Handgrip Strength

After participants became familiar with the equipment, they remained seated, with their shoulders in neutral position, elbows flexed at 90°, and fist in neutral position. Subjects were instructed to perform a maximum isometric contraction. Three attempts were made with alternate limbs, with a 60-second interval between attempts. The highest achieved reading was recorded for subsequent analyses. No verbal encouragement was offered during the test [21].

### 2.5. Timed Up and Go Test (TUG)

A stopwatch was used to measure the time spent to get up from a chair, walk a 3-meter distance on habitual speed, go around an obstacle, and sit again. We used a chair with a 45-inch tall seat, a 65-inch tall armrest, and a full, straight backrest [22].

### 2.6. Sitting and Lift Test

The sitting and lift test was conducted in a chair with a 45 cm tall seat but with no lateral, armrest support and with a full, straight backrest leaning on a wall. Each time a participant stood up, it was counted out loud, and five repetitions were timed [23]. There was a period of familiarization to ensure that the participant's sitting position occupied the largest part of the seat.

### 2.7. Mini-Mental State Examination (MMSE)

Answers were obtained from questions presented in the interview format, and a score cut was set based on the scores of 13 points for illiterate people, 18 points for people with 1 to 8 years of schooling, and 26 points for those with more than 8 years of schooling [24, 25].

### 2.8. Depression and Functionality Levels

The Katz scale was used to assess the autonomy of the elderly to perform the basic activities of daily living, with conditions classified according to scores achieved as total (0 point), severe (1 or 2 points), moderate (3 or 4 points), or light dependency (5 points) as well as independent (6 points). The scale of Yesavage was used for the screening of depression, assessing cognitive and behavioral aspects typically affected among older adults. In the present study, 11 to 15 points accounted for severe depression, whereas 6 to 10 points for mild depression and 0 to 5 as indicative of no depression. The Katz and Yesavage scales were implemented as described elsewhere [26].

### 2.9. Blood Drawing and Testing

Blood samples were obtained through a venous puncture. Serum triglycerides (TGs), high-density lipoprotein (HDL-c), total cholesterol (TC), and glucose were analyzed by enzyme-based colorimetric methods using commercially available kits (Advia 2400, SIEMENS Healthcare Diagnostics Inc., Tarrytown, USA). The Friedewald equation was used to yield low-density lipoprotein (LDL-c) and very low-density lipoprotein (VLDL-c) estimates [27]. Serum insulin was determined by fluoroimmunoassay using a commercially available kit (Immulite 2000, SIEMENS Healthcare Diagnostics Inc., UK). Vitamin D_3_ (25 hydroxyvitamin D_3_) was analyzed by double antibody radioimmunoassay using Vit D25 preextraction with acetonitrile (DiaSorin Inc., Stillwater, USA). Inflammatory mediators were analyzed using the enzyme-linked immunosorbent assay (ELISA) method with specific kits for each cytokine (eBioscience, USA). High-sensitivity C-reactive protein was determined by immunonephelometry (CardioPhase, Dade Behring, USA).

### 2.10. Identification of Frailty

Frailty was identified based on criteria described by Fried et al., which include unintentional weight loss, reports of exhaustion, reduction of walking speed, muscle weakness, and low level of physical activity. Elderly who did not display any of the criteria mentioned above were classified as nonfrail, whereas patients fitting in 1 or 2 criteria were considered prefrail. Only elderly displaying 3 or more criteria were classified as frail.

Loss of more than 4.5 kg or 10% of the body weight over the last year was considered as weight reduction. Exhaustion was identified when there was self-reported fatigue. Low walking speed was identified by measuring the time required to walk a distance of 4.0 meters forward and return. Males of height of <1.73 and ≥1.73 meters and females of height of <1.59 and ≥1.59 meters tested positive by reaching times of >7 and >6 seconds, respectively. Muscle weakness was defined on the basis of the handgrip strength test. Males scored positive by displayed strengths of <29.0 kgf for BMI < 24.0 kg/m^2^, or <30.0 kgf for BMI 24.1–26.0 kg/m^2^, or <32.0 kgf for BMI > 26.0 kg/m^2^, while positive female patients were those with values of 17.0 < kgf for BMI < 23.0 kg/m^2^, or <17.3 kgf for BMI 23.1–26.0 kg/m^2^, or <18.0 kgf for BMI 26.1–29.0 kg/m^2^, or <21.0 kgf for BMI > 29.0 kg/m^2^. Patients scored for low level of physical activity when declared exercising with a frequency lower than twice a week.

### 2.11. Physical Exercise Program

The multiple component exercise program lasted 12 weeks, being performed 3 times per week with each session lasting 40 minutes. Exercises were focused on improving mobility, flexibility, strength, and aerobic resistance (Table 1). Physical fitness and exercises for the training program were chosen after an initial assessment of the volunteers to gather variables to improve the independence of elderly people.

To implement the program, the EG was divided into 3 subgroups of approximately 5 elderly people in each group so that exercises were conducted in a personalized and individualized way. The subgroup was followed at all times by 2 physical educators and 3 monitors who did not know which elders were in each of the groups. The intensity and complexity of exercises were defined based on the initial assessment and adjusted weekly considering the subjective perception of effort (SPE) reported by each participant.

Owing to the clinical condition of participants, characterized by a high level of functional impairment, medical clearance was granted to perform up to moderate intensities of exercise, what resulted in exercises between 5 and 7 of SPE being selected according to an adapted Omni scale [28]. The intensity was collected during the exercise sessions during the 12 weeks.

### 2.12. Statistical Analysis

The sample size was estimated based on an “a priori” calculation, using the dependent variables “TUG” and “handgrip strength.” After the analysis of preliminary data, with an alpha of 0.05 and a power of 0.80 for a two-tailed test, and assuming uneven groups, a sample of at least 12 participants for the EG and of 18 for the CG would be required to detect a significant difference in the order of 50% between treatment arms.

Clinical, anthropometric, biochemical, and inflammatory parameters on the postintervention moment were compared between the groups (intervention and control) using analysis of covariance (ANCOVA) models for variables with Gaussian distribution. Nevertheless, the nonparametric ANCOVA was employed for the variables that did not display Gaussian distribution in at least one of the groups. For ANCOVA models, measures obtained during postintervention were deemed dependent variables, whereas the groups (exercise and control) were deemed independent variables, with baseline measures as covariates. Intragroup comparisons of measurements were performed using the Student's *t*-test for paired samples or using a nonparametric Wilcoxon test whether measures displayed a Gaussian distribution or not, respectively. Comparisons of mean measurements between groups were conducted by the independent *t*-test. Comparisons of frequencies between groups used the chi-square test. A *p* value of less than 0.05 was considered significant. Analyses were carried out using SAS v 9.4 (SAS Institute, Inc., 1999).

## 3. Results

EG participants had a 61.5% attendance to exercise sessions and reported an average SPE of 5.2 during intervention. Tables 2 and 3 display descriptive statistics of clinical, anthropometric, biochemical, functional, and inflammatory variables observed in the sample, with their respective intragroup and intergroup comparisons.

Functional performance variables significantly improved in the EG when baseline and postintervention values were compared. The EG showed statistically superior left and right handgrip strength measures by 33 and 26%, respectively, in comparison to the CG endpoint levels. Regarding TUG and stand up/sit down tests, the EG had statistically lower values than the CG (38% for TUG and 29% for stand up/sit down). Also in the EG, anthropometric traits and MMSE exhibited no significant differences when baseline and postintervention were compared. None of these variables in the CG presented differences between the baseline and postintervention moments.

Regarding the biochemical and inflammatory traits, values checked for glucose, insulin, total cholesterol, triglycerides, vitamin D_3_, and CRP showed significant differences when comparing baseline and postintervention moments in the EG, while only glucose values differed in the CG. No other changes were observed concerning biochemical and inflammatory variables among groups or moments.

**Figure 2:**
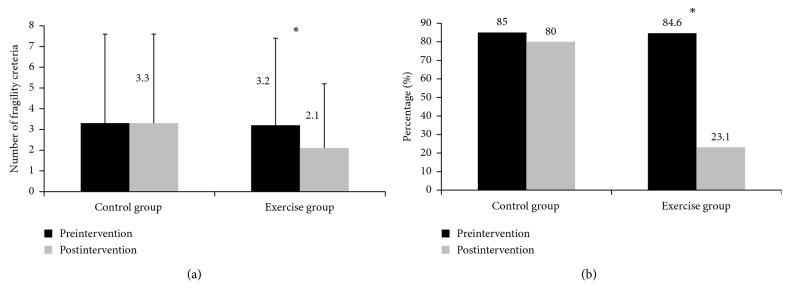
Absolute count of criteria for frailty (a) and proportion of elderly deemed frail and prefrail (b) in the control group and the exercise group. Black bar: preintervention moment; gray bar: postintervention moment. ^∗^*p* < 0.05.

Figure 2 shows the absolute prevalence of criteria for frailty as well as frequencies of elderly classified as frail and prefrail in pre- and postintervention moments. The EG showed *a* ≈ 34% reduction in the prevalence of criteria for frailty, while the CG showed a reduction of ≈6% between evaluations conducted before and after the exercise program. A decrease of more than ≈73% in the number of elderly classified as frail was noted in the EG, with most participants migrating to a prefrail status. On the other hand, the CG remained similar between pre- and postintervention periods.

## 4. Discussion

The main findings of this study showed an improvement in the biochemical and functional capacity (strength, speed, and agility) of residents of long-term care institutions after undertaking a 12-week exercise training program. Improvements in biochemical, anthropometric, or inflammatory variables were not apparent when crude endpoints values of the exercise and control groups were compared. However, traits such as serum glucose, insulin, total cholesterol, triglycerides, vitamin D_3_, and CRP showed significant improvements when baseline and postintervention stages of the EG are compared. Furthermore, a reduction in the absolute count of criteria elements for frailty was observed as well as in the proportion of the elderly considered frail in the EG after intervention.

The baseline physical status of the elderly participants presented extremely low performance scores when compared to studies conducted elsewhere, rending a sample with a major dysfunctional status [29–32]. Exercise-induced improvements in physical performance of institutionalized older adults are corroborated by previous reports [19], resulting in decreased risk of falls and attenuation of frailty. Nevertheless, Faber et al. reported the effects of 2 exercise programs with moderate intensity conducted in 15 long-term care institutions and found that both programs had positive effects on physical performance in prefrail elderly people but not in frail individuals [33]. On the other hand, the intervention described in the present study was successful in reversing the frailty phenotype in more than 70% of the frail elderly [34].

Also, a systematic review of randomized clinical trials with frail elderly patients showed contradictory data regarding improvements in risk for falls, mobility, balance, strength, functional capacity, and body composition [20]. Even among those who have shown improvements in functional capacity, it is not appropriate to attribute the success of these results to a specific element of the intervention protocol. Although most studies usually describe the intervention program in detail, some reports fail to describe appropriately load-related parameters, such as heart rate, lactate concentration, or SPE during interventions, and that might at least in part contribute to the contradictory results in the literature.

The mean PSE of 5.2 obtained herein renders the intensity of our exercise as moderate and supports a beneficial effect of this exercise intensity on functional parameters [35–37], risk of falling [33], balance [38], and self-reported health status [39]. Other studies show that exercise programs with higher intensity seem to generate better results [40, 41]. In this context, we believe that, besides intensity, other variables such as the duration, method, and type of the exercise may influence results in studies involving exercise programs in frail elderly, since responsiveness to training, time, and history of exposure cannot be neglected.

The literature shows that participation of the elderly population in regular physical activity is considered protective against various components of frailty [42]. Moreover, physical fitness among aged individuals is associated with better functioning of glucose and lipid metabolisms, with greater lean mass as well as with a lower, systemic inflammation [9, 11, 12, 43]. However, the physical training program conducted herein was not efficient in promoting improvements in the investigated interleukins and anthropometric variables. This suggests that the intervention was effective but limited to promoting adaptations on systems directly related to functionality (mainly neuromuscular effects) and on few metabolic traits (glucose, insulin, total cholesterol, triglycerides, vitamin D_3_, and CRP). One possible explanation for such a narrowed repercussion can be based on the low adherence to our exercise program (61.5%). For instance, the Brazilian Guidelines for Cardiac Rehabilitation [44] affirm that patients who firmly adhere to programs display more pronounced adaptations in hemodynamic, metabolic, myocardial, vascular, and psychological parameters, not observed in the conditions herein. Another study analyzed the association between adherence and physiological changes in middle-aged adults, concluding that high adherence promoted improvements in physical parameters and quality of life [45]. In general, the literature presents consistent evidence that adherence to the exercise program associates with improved clinical outcomes, functional performance, and quality of life [46], and the levels of attendance described herein can be rendered as a limitation of the study.

Thus, more studies testing models of physical exercise with institutionalized elderly people are necessary to yield more beneficial protocols, having in mind protocols already in literature that were successful in reducing systemic inflammation and regulating the metabolic profile to boost anabolism and muscle protein synthesis [13]. But any intervention should take into consideration individual aspects. The type, intensity, and duration of the exercise program must be carefully chosen, and the frailty status, medical recommendations, history of diseases, and physical activity should be considered. In this case, programs based on multiple components, which address physical qualities such as mobility, balance, flexibility, aerobic capacity, and strength, similar to the model used in this study may be used. Part of literature supports this type of intervention, stating that these programs are more effective at improving general health of frail elderly people [19, 30, 47].

The present study is not without limitations. For example, a low participation rate in the exercise sessions was noted. Despite constant stimulation and customized work, adherence to the program was below expectations. Another limitation was the extremely low physical conditioning of the participants, as well as the elevated prevalence of depression, which explains greater social isolation and lower adherence to the exercise program, even though, at the end of the exercise program, there was a significant reduction in depression rates.

Nevertheless, our results corroborate that, regardless of physical limitations and frailty severity, elderly people are capable of participating in personalized training programs when their limits are considered. Additionally, strength training must be taken into account and should be carried out in an adapted, assisted way [32]. Training should offer benefits even when it is carried out in a nonintensive way. In addition, physical exercises can be a very effective strategy to implement beneficial routines to residents of long-term care institutions, particularly those with greater impairment as well as those diagnosed with frailty.

## 5. Conclusion

This study showed that a 12-week exercise training program in elderly patients with the syndrome of frailty living in long-term care institutions was efficient in improving aspects of their functional capacity, including muscle strength, speed, and agility, as well as glucose, insulin, total cholesterol, triglycerides, vitamin D_3_, and CRP. Moreover, the intervention helped to reduce the amount of criteria for frailty syndrome, and there was a reversal of the frailty condition in a considerable number of elderly who attended the program. However, there was no repercussion regarding anthropometric, biochemical, and inflammatory features.

## Figures and Tables

**Table 1:** Schematic demonstration of the weekly physical training program.

	Physical training program (weeks/time)											
Variables	1 W	2 W	3 W	4 W	5 W	6 W	7 W	8 W	9 W	10 W	11 W	12 W
Mobility	20′	20′	15′	15′	—	—	—	—	—	—	—	—
Flexibility	—	—	10′	10′	10′	10′	10′	10′	—	—	—	—
Aerobic resistance	20′	20′	15′	15′	15′	15′	10′	10′	15′	15′	10′	10′
Strength training	—	—	—	—	15′	15′	20′	20′	25′	25′	30′	30′
Total time	40′	40′	40′	40′	40′	40′	40′	40′	40′	40′	40′	40′

**Table 2:** Comparison of anthropometric, clinical, and functional performance variables between baseline and postintervention moments.

Variable	Exercise group (*n* = 13)		Control group (*n* = 24)	
	Baseline	Postintervention	Baseline	Postintervention
Age (years)	73.3 ± 6.4	—	77.8 ±8.0	—
Weight (kg)	67.4 ± 10.1	—	66.7 ± 11.9	—
Height (m)	1.59 ± 9.7	—	1.59 ± 8.6	—
Diseases (number)	2.2	—	1.7	—
Depression (%)	28.6%	18.2%^∗^	24.0%	22.7%
Dependence (%)	36.4%	0.0%^∗^^‡^	22.7%^§^	20.8%
MMSE (points)	17.4 (13.8; 20.9)	18.3 (15.8; 20.9)	18.1 (15.3; 21.0)	17.0 (15.1; 18.9)
BMI (kg/m^2^)	26.4 (23.6; 29.2)	27.0 (26.4; 27.6)	26.8 (25.1; 28.6)	26.5 (26.1; 27.0)
Conicity index	1.36 (1.32; 1.40)	1.36 (1.34; 1.38)	1.38 (1.35; 1.41)	1.39 (1.37; 1.40)
Waist (cm)	95.8 (89.9; 101.6)	97.0 (95.2; 98.8)	97.96 (93.4; 102.5)	97.84 (96.4; 99.3)
LHS (kgf)	8.7 (3.7; 13.7)	16.7 (14.7; 18.7)^†&^	10.5 (8.1; 13.0)	11.2 (9.5; 12.9)
RHS (kgf)	10.1 (5.9; 14.4)	17.2 (15.2; 19.2)^†#^	11.0 (7.6; 14.4)	12.7 (11.0; 14.4)
TUG (s)	28.8 (20.4; 37.2)	20.9 (18.3; 23.4)^†&^	29.1 (21.3; 36.9)	28.9 (26.6; 31.2)
Stand/sit time (s)	33.5 (22.0; 45.0)	20.3 (17.2; 23.4)^†#^	27.7 (23.0; 32.5)	26.1 (23.6; 28.7)

^∗^ *p* < 0.05 for intragroup comparisons calculated using the chi-square test; ^‡^*p* < 0.05 for comparison of postintervention measurements between groups (exercise group versus control group) calculated using the chi-square test; ^§^*p* < 0.05 for comparison of baseline measurements between groups (exercise group versus control group) calculated using the chi-square test; ^†^*p* < 0.01 for intragroup comparisons calculated using the paired *t*-test or Wilcoxon test. ^#^*p* < 0.01; ^&^*p* < 0.01 for comparison of postintervention measurements between groups (exercise group versus control group) calculated using ANCOVA or nonparametric ANCOVA, adjusted by base measurements as a covariate. MMSE = mini-mental state examination; BMI = body mass index; LHS = left handgrip strength; RHS = right handgrip strength; TUG = timed up and go test.

**Table 3:** Comparison of metabolic and inflammatory variables between baseline and postintervention moments.

Variable	Exercise group (*n* = 13)		Control group (*n* = 24)	
	Baseline	Postintervention	Baseline	Postintervention
Glycaemia (mg/dl)	100.9 (86.9; 115.0)	95.7 (88.3; 103.0)^†^	105.0 (84.6; 125.4)	94.3 (89.1; 99.5)^‡^
Insulin (*µ*I/ml)	14.9 (10.1; 19.7)	11.3 (6.1; 16.4)^†^	16.0 (10.9; 21.1)	12.9 (9.3; 16.5)
TC (mg/dl)	168.5 (144.9; 192.0)	148.0 (132.9; 163.2)^‡^	166.4 (151.1; 181.7)	165.8 (155.1; 176.5)
TR (mg/dl)	151.7 (119.1; 184.3)	105.7 (83.7; 127.6)^†^	120.1 (93.0; 147.3)	120.7 (105.5; 136.0)
HDL (mg/dl)	48.6 (41.5; 55.7)	49.1 (44.4; 53.8)	50.3 (45.6; 54.9)	45.8 (42.5; 49.1)
LDL (mg/dl)	87.9 (72.8; 103.1)	83.5 (72.0; 95.0)	89.3 (76.4; 102.3)	93.7 (85.5; 101.8)
Vitamin D_3_ (ng/ml)	21.7 (17.3; 26.2)	26.0 (22.6; 29.5)^‡^	22.2 (19.2; 25.1)	23.9 (21.4; 26.4)
CRP (mg/dl)	0.68 (0.46; 0.90)	2.41 (1.48; 3.35)^‡^	2.07 (−0.58; 4.7)	1.67 (0.98; 2.4)
IL6 (pg/ml)	14.0	17.1 (14.39; 19.86)	18.0 (14.7; 21.3)	16.1 (14.0; 18.2)
IL10 (pg/ml)	13.0	15.6 (11.4; 19.7)	17.9 (14.3; 21.5)	16.3 (13.0; 19.5)
IL1a (pg/ml)	14.6 (10.7; 18.4)	16.0 (14.0; 18.0)	17.6 (13.9; 21.4)	16.8 (15.23; 18.4)
IL1RAcP (pg/ml)	18.9 (12.9; 25.0)	16.1 (10.6; 21.6)	15.1 (10.7; 19.4)	16.7 (12.5; 21.0)

^‡^ *p* < 0.05; ^†^*p* < 0.01 for intragroup comparisons calculated by the paired *t*-test or Wilcoxon test. TC = total cholesterol; TR = triglycerides; HDL = high-density lipoprotein; LDL = low-density lipoprotein; CRP = C-reactive protein; IL = interleukin.
